# Network pharmacology and computer-aided drug design to explored potential targets of Lianhua Qingwen and Qingfei Paidu decoction for COVID-19

**DOI:** 10.3389/fphar.2022.1013428

**Published:** 2022-09-23

**Authors:** Liyuan Li, Xiaoying Wang, Xiao Guo, Yikun Li, Qiuhang Song, Aiying Li

**Affiliations:** ^1^ College of Basic Medicine, Hebei University of Chinese Medicine, Shijiazhuang, China; ^2^ Hebei Key Laboratory of Chinese Medicine Research on Cardio-Cerebrovascular Disease, Hebei University of Chinese Medicine, Shijiazhuang, China; ^3^ Hebei Higher Education Institute Applied Technology Research Center on TCM Formula Preparation, Shijiazhuang, China

**Keywords:** COVID-19, traditional Chinese medicine, systems biology, GRM1/5, arctiin, β-carotene

## Abstract

Coronavirus disease 2019 (COVID-19) caused by SARS-CoV-2, has spread globally, affecting people’s lives worldwide and hindering global development. Traditional Chinese Medicine (TCM) plays a unique role in preventing and treating COVID-19. Representative prescriptions for the COVID-19 treatment, Lianhua Qingwen (LHQW) and Qingfei Paidu Decoction (QFPD), effectively alleviate COVID-19 symptoms, delaying its progression and preventing its occurrence. Despite the extensive similarity in their therapeutic effects, the mechanisms and advantages of LHQW and QFPD in in treating mild-to-moderate COVID-19 remain elusive. To characterize the mechanisms of LHQW and QFPD in treating COVID-19, we used integrated network pharmacology and system biology to compare the LHQW and QFPD components, active compounds and their targets in Homo sapiens. LHQW and QFPD comprise 196 and 310 active compounds, some of which have identical targets. These targets are enriched in pathways associated with inflammation, immunity, apoptosis, oxidative stress, etc. However, the two TCM formulas also have specific active compounds and targets. In LHQW, arctiin, corymbosin, and aloe-emodin target neurological disease-related genes (GRM1 and GRM5), whereas in QFPD, isofucosterol, baicalein, nobiletin, oroxylin A, epiberberine, and piperlonguminine target immunity- and inflammation-related genes (mTOR and PLA2G4A). Our findings indicate that LHQW may be suitable for treating mild-to-moderate COVID-19 with nervous system symptoms. Moreover, QFPD may effectively regulate oxidative stress damage and inflammatory symptoms induced by SARS-CoV-2. These findings may provide references for the clinical application of LHQW and QFPD.

## Introduction

Coronavirus disease 2019 (COVID-19) was first reported at the end of 2019. It then spread into a severe global public health pandemic, causing irreparable harm to human health. Severe acute respiratory syndrome coronavirus 2 (SARS-CoV-2) is the causative pathogen of COVID-19. Patients with mild-to-moderate COVID-19 often present cough, fever, muscle pain, and diarrhea, while patients with severe disease often present a strong inflammatory response and multiple organ failure. In fact, some patients exhibit central nervous system dysfunction and olfactory sensory disorder (Asadi-Pooya & Simani, 2020; Havervall et al., 2021; Nguyen et al., 2022). Moreover, recovered patients often experience sequelae, such as dyspnea and fatigue (Carfì et al., 2020). Various therapeutic strategies have been developed for treating COVID-19, including those derived from Traditional Chinese Medicine (TCM) (Salian et al., 2021). Among these TCM therapies, Lianhua Qingwen (LHQW) and Qingfei Paidu decoction (QFPD) in “Three Chinese Patent Medicines and Three TCM Prescriptions” were commonly used with high efficacy (Zhang S. et al., 2022).

Moreover, the components in LHQW capsules, namely forsythia, honeysuckle, isatis root, and herbal houttuynia, have properties that help heat-clearing, detoxification, and lung ventilation, which are significant in the treatment of influenza caused by the influenza A virus infection (Li L. C. et al., 2020; Gao et al., 2020; Zhuang et al., 2020; Chu et al., 2021). In addition, QFPD comprises four classic Chinese medicine prescriptions, namely Shegan Mahuang decoction, Maxing Shigan decoction, Xiaochaihu decoction, and Wuling powder. QFPD contains 21 main components, namely Belamcandae rhizoma, Alisma orientale, and Polyporus umbellatus, among others.

QFPD is widely used throughout China (Liu et al., 2020) for treating COVID-19 and has demonstrated efficacy in preventing disease progression, improving clinical symptoms, alleviating lung lesions, and reducing mortality rates, without inducing severe adverse effects (Zhang L. et al., 2022). Additionally, QFPD plays roles in inflammation, immunoregulation, neuroprotection, and lung injury reduction, among other physiological processes (Chen et al., 2020). Although both LHQW and QFPD can be used to treat mild-to-moderate COVID-19, their components are distinct, indicating that the similarity and specificity of the biological mechanisms underlying the effects of the two formulations warrant further investigation using contemporary platforms.

Previous studies demonstrated that patients with mild-and-moderate COVID-19 often show cough, inflammation, immune response, nervous system dysfunction, etc. To reveal the mechanism of LHQW and QFPD in treating these symptoms, we compared the LHQW and QFPD components, active compounds, and their targets in *Homo sapiens*. We found that LHQW and QFPD contain 196 and 310 active compounds respectively, they have common and specific targets. The common targets are enriched in pathways associated with inflammation, immunity, apoptosis, and oxidative stress. The special active compounds (Arctiin, Corymbosin, and Aloe-emodin) of LHQW target neurological disease-related genes (GRM1 and GRM5), and the special active compounds (isofucosterol, baicalein, nobiletin, oroxylin A, epiberberine, and piperlonguminine) of QFPD target immunity- and inflammation-related genes (mTOR and PLA2G4A). This finding suggested that LHQW may improve mild-to-moderate COVID-19 with nervous system symptoms, whereas QFPD may potentially regulate oxidative stress damage and inflammatory symptoms. These findings may provide potential references for the clinical application of LHQW and QFPD. Collectively, our study provides theoretical support for the effective administration of combinatorial TCM and allopathic medicine for the treatment of COVID-19.

## Materials and methods

### Selection and analysis of LHQW and QFPD active compounds

The composition, channel tropism, properties, and flavors of all LHQW and QFPD components were obtained from the Chinese Pharmacopoeia (2015 edition) (Commission, 2015). The active compounds with oral bioavailability (OB) ≥ 30% and drug-likeness (DL) ≥ 0.18 were derived from the Traditional Chinese Medicine Systems Pharmacology Database (TCMSP, http://tcmspw.com/tcmsp.php) (Ru et al., 2014).

### Acquisition of common and specific active compounds targets in LHQW and QFPD

Representative genes targeted by LHQW and QFPD active compounds were identified from the TCMSP database. To compare the similarity and specificity of LHQW and QFPD, we constructed a Venn diagram of target genes to determine their common and specific targets.

### Construction of a protein-protein interaction (PPI) network and pathway enrichment analysis

The STRING (https://string-db.org/) (Szklarczyk et al., 2021) database was used to construct a PPI network of common targets, which was then topologically analyzed to identify hub targets. The targets in the PPI network were screened by “degree,” “between centrality,” and “closeness centrality,” and targets with scores greater than the median of the three parameters were selected to build a protein-protein interaction network (PIN). The molecular complex detection (MCODE) algorithm was used in the Cytoscape software to obtain the protein modules. In addition, the KOBAS (http://kobas.cbi.pku.edu.cn/) (Xie et al., 2011) database was used for Gene Ontology (GO) and Kyoto Encyclopedia of Genes and Genomes (KEGG) pathway enrichment. The most enriched GO and KEGG pathways were extracted to analyze and compare the similarities between LHQW and QFPD.

### Analyzing LHQW and QFPD specificity

The TCMSP database was used to identify active compounds that could target specific genes, as well as the TCM components that contained these active compounds. To determine the relationship between TCM components, active compounds, and specific targets, “TCM component–active compound–specific target” networks were constructed using Cytoscape software. The MCODE algorithm of the Cytoscape software was used to build the protein modules of specific genes in the networks.

### Molecular docking analysis

The most significant target genes of LHQW and QFPD genes were selected, and the structures of proteins encoded by the target genes were obtained from the RCSB Protein Data Bank (RCSB PDB, https://www.rcsb.org/). The 3D structures of the active compounds were obtained from PubChem (https://pubchem.ncbi.nlm.nih.gov/). GOLD 5.2 and Molecular Operating Environment v2019.0102 (MOE) software were used for virtual docking, and ChemScore, ChemPLP and binding energy value were used in combination to evaluate the docking results. Finally, docking results were visualized using PyMol 1.8 and MOE.

### Molecular dynamics simulation

After molecular docking, Amber 18 software was used to simulate the molecular dynamics of the targets and active compounds of the TCM preparation. Using Amber 99sb force parameters, the system was executed for 50 ns. Every 10 ps, trajectory data were saved with a 2-ps time step. The Gibbs free energy of the active compounds and proteins was calculated using the molecular mechanics/Poisson-Boltzmann surface area (MM/PBSA) method. The results were estimated using Gibbs free energy, root mean square deviation (RMSD), root mean square fluctuation (RMSF), and solvent-accessible surface area (SASA) metrics.

## Results

### Comparison of LHQW and QFPD composition, properties, flavors, and channel tropism

**FIGURE 1:**
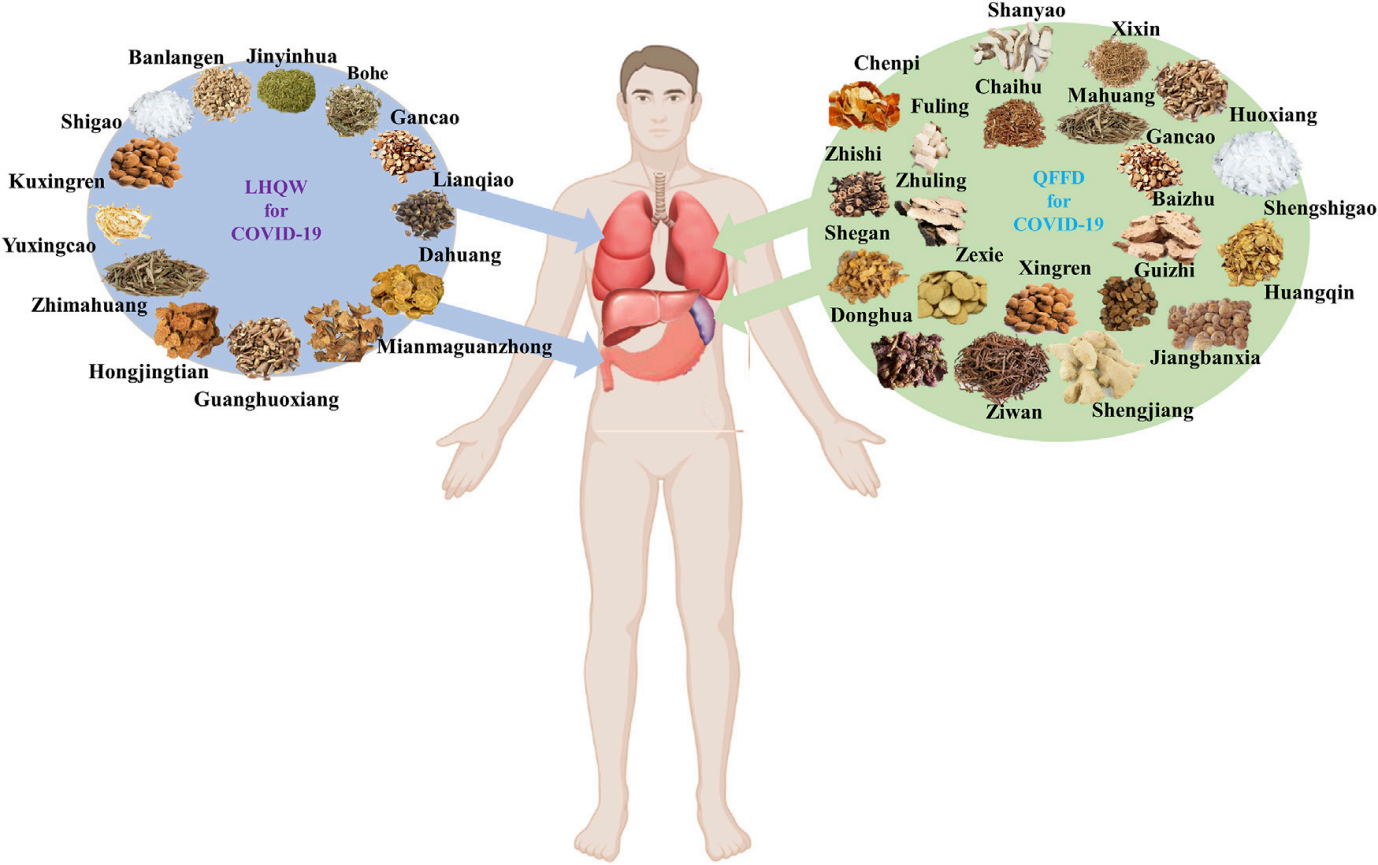
Main channel tropism of Lianhua Qingwen (LHQW) and Qingfei Paidu Decoction (QFPD) based on the TCM theory.

LHQW and QFPD components, properties, flavors, and channel tropism, are listed in Supplementary Table S1. Based on the TCM principle that most components in LHQW have “cold properties,” this formulation can be considered a “cold medicine”. In contrast, QFPD could be regarded as a ‘warm medicine’ as most of its components possess “warm properties.” Channel tropism is a critical TCM concept in which a drug can exhibit selective action in a specific part of the human body. Eight of the thirteen components in LHQW act through the lung channel, while the remaining five components act through the stomach channel. Nine of the twenty-one components in QFPD act through the lung channel, while nine belong to the spleen channel. Collectively, LHQW seemingly exerts a heat-clearing effect primarily through the lung channel, whereas QFPD exerts a dampness-eliminating effect primarily through the lung channel (Figure 1).

### Comparison of LHQW and QFPD active components and their potential targets

LHQW contained 196 active compounds that targeted 249 genes, while QFPD contained 310 active compounds that targeted 261genes (Supplementary Table S2). The constructed Venn diagrams of the LHQW and QFPD active compounds and their targets identified 135 common compounds, while the other compounds were unique to either formula (Supplementary Figure S1A). Moreover, LHQW and QFPD had 226 common targets, whereas LHQW and QFPD targeted 23 and 35 specific targets, respectively (Supplementary Figure S1B). These data suggest that the two formulations may exert similar effects in treating mild-to-moderate COVID-19. The two prescriptions may contain the same components, or different components with the same active compounds, different active compounds with the same targets. However, they also have certain unique compounds and targets, thereby eliciting varying effects in the treatment of mild-to-moderate COVID-19.

### PPI analysis of the common active compound targets in LHQW and QFPD

**FIGURE 2:**
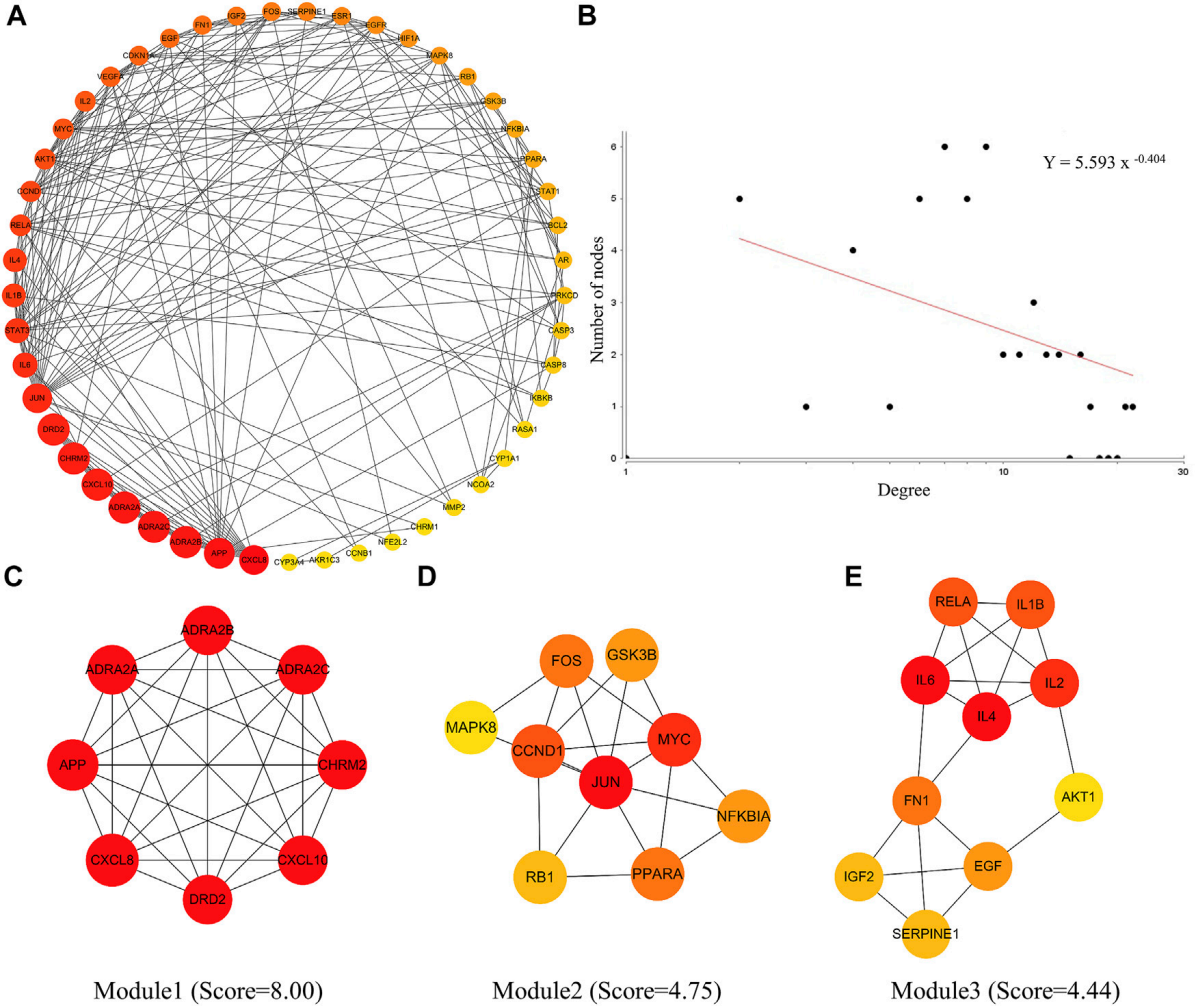
Protein interaction network (PIN) construction and network topology analysis. **(A)** PIN was constructed using the common host targets of LHQW and QFPD. **(B)** PIN topology analysis was performed using the Network Analyzer of the Cytoscape software. **(C–E)** The significant module was identified from the PIN using the molecular complex detection (MCODE) method with a score >4.0; the red and yellow nodes represent hub genes, yellow represents the lower degree and red represents the higher degree.

After entering the 226 common targets into the STRING database, we constructed a PPI network, and 49 hub targets were selected to build the PIN (Figure 2A). The results of the PIN topology analysis (Figure 2B) showed that it possessed the robustness and vulnerability topologies required for network research (*γ* = 0.404, *γ* < 3), and these targets could be considered hub targets. Following the biological functional clustering of targets in the PIN, three protein modules (score >4) were obtained (Figures 2C–E). These were believed to play an important role in network regulation and may be related to immune function and inflammatory responses. These results indicate that LHQW and QFPD may play comparable roles in treating mild-to-moderate COVID-19 by regulating the protein modules in the PIN.

### GO and KEGG pathway enrichment analysis of common targets

**FIGURE 3:**
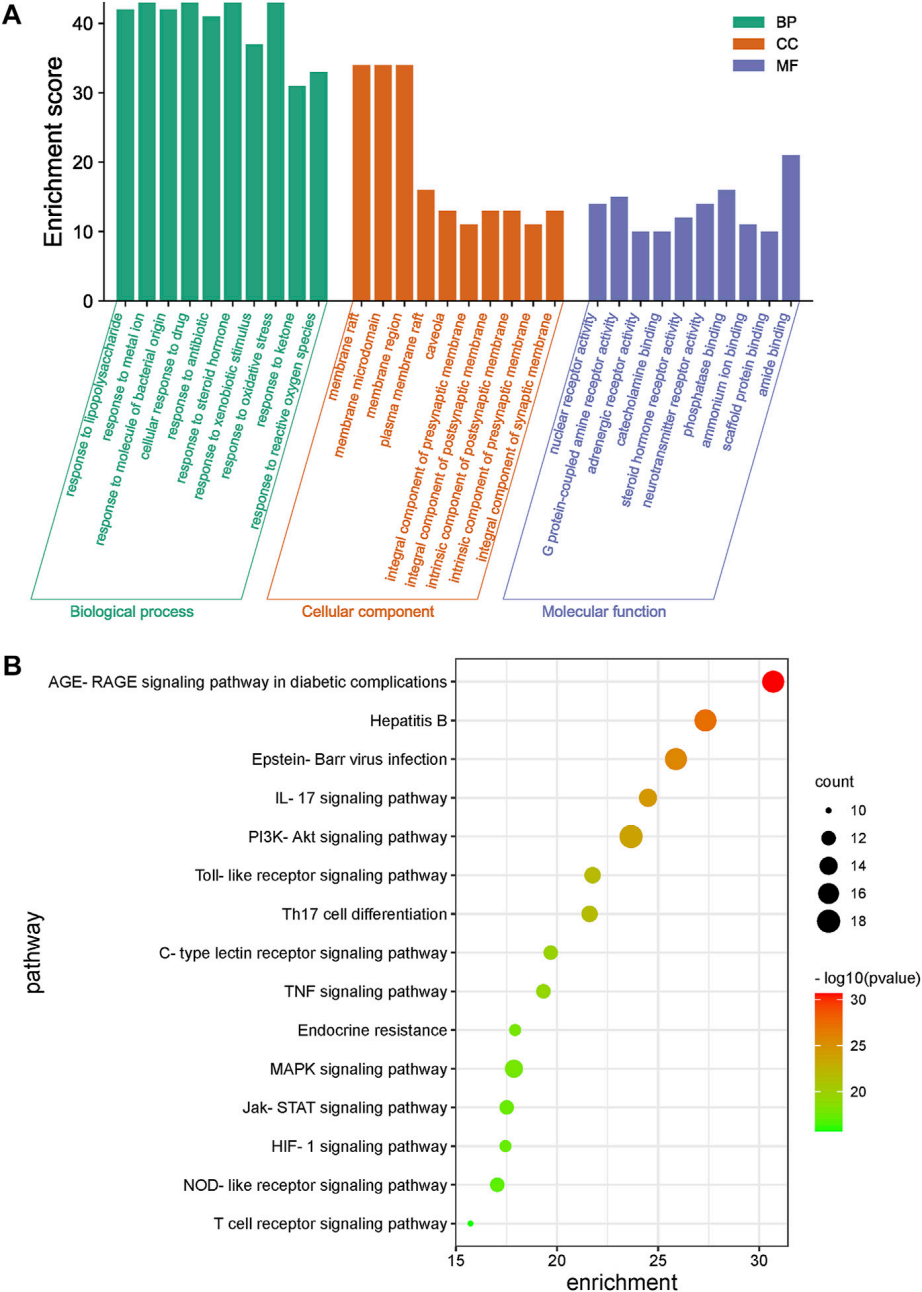
Gene Ontology (GO) and KEGG enrichment analysis of common genes of LHQW and QFPD targeted during the treatment of mild-to-moderate COVID-19. **(A)** GO analysis was performed for the top 10 GO terms identified; *X*-axis represents GO term categories, while the *y*-axis represents the enrichment score. **(B)** A KEGG analysis of the genes was performed, and the top 15 pathways are shown. The color of the dot represents the *p*-value. The dot size represents the number of target genes enriched in the pathway.

The GO enrichment results for the 49 key common genes targeted by LHQW and QFPD (Figure 3A) showed that both formulations might have roles in inflammation, immunity, energy metabolism, and oxidative stress through various biological pathways, such as cytokine activity and responses to xenobiotic stimuli, nutrient levels, and oxidative stress. KEGG enrichment results further showed that the signaling pathways of the two formulations were primarily associated with various viral infections (hepatitis B, Epstein-Barr virus), inflammatory reactions (advanced glycation end-product [AGE]-receptor for AGE [RAGE] signaling pathway involved in diabetic complications, IL-17 signaling pathway), and immunoregulation (Toll-like receptor signaling pathway, PI3K-Akt signaling pathway). These results suggest that LHQW and QFPD may slow the progression of mild and moderate COVID-19 by regulating similar mechanisms, including inflammation, multiple viral infections, immunoregulation, energy metabolism, and oxidative stress (Figure 3B).

### Analysis of LHQW and QFPD specificity

**FIGURE 4:**
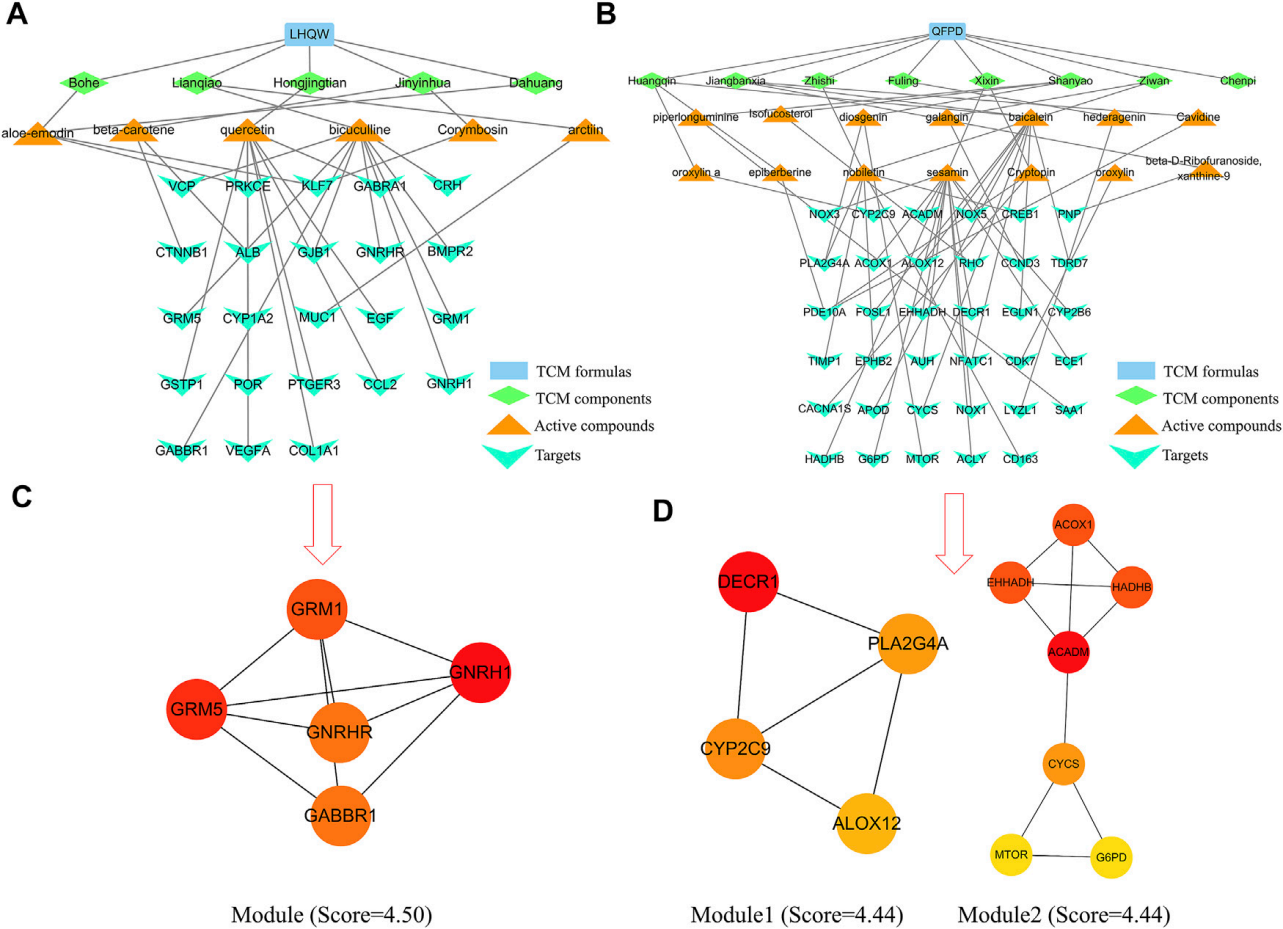
“TCM component–active compound–specific target” networks and protein modules of LHQW and QFPD. The green diamond represents the TCM components, the orange triangle represents the active compounds, and the cyan fusiform represents the network targets. **(A)** Interconnection between TCM components, active compounds, and the specific targets of the active compounds in LHQW. **(B)** Interconnection between TCM components, active compounds, and their specific targets of the active compounds in QFPD. **(C)** The significant module identified from the specific targets of LHQW. **(D)** The significant module identified from the specific targets of QFPD.

Table 1 lists the active compounds and their specific targets obtained from the TCMSP database. After importing the relationship between TCM components as well as active compounds and their specific targets into the Cytoscape software, the “TCM component–active compound–specific target” networks (Figures 4A–D) and three protein modules (score ≥4.44) of specific LHQW and QFPD targets were constructed (Figures 4C,D). These modules suggest that metabotropic glutamate receptors GRM1 and GRM5 play a vital role in conduction and neural network regulation.

**TABLE 1:** Specific targets of the active compounds present in LHQW and QFPD.

TCM formula	Active compounds	Specific targets
LHQW	Aloe-emodin, Arctiin, Bicuculline, Beta-carotene, Corymbosin, Quercetin	MUC1, GABBR1, BMPR2, GRM5, GNRH1, GNRHR, CRH, GJB1, GRM1, VCP, PRKCE, KLF7, GABRA1, VEGFA, EGF, POR, CYP1A2, CCL2, PTGER3, COL1A1, GSTP1, ALB, CTNNB1
QFPD	Nobiletin, Hederagenin, Baicalein, Oroxylin a, Epiberberine, Cavidine, Isofucosterol, Diosgenin, Cryptopin, Sesamin, Galangin, xanthine-9, Piperlonguminine, Beta-d-Ribofuranoside	LYZL1, FOSL1, CYCS, NFATC1, TDRD7, EGLN1, NOX5, APOD, CDK7, CYP2C9, PDE10A, PNP, CCND3, RHO, CACNA1S, G6PD, ECE1, ACADM, CYP2B6, NOX3, NOX1, ACOX1, ACLY, EHHADH, AUH, HADHB, SAA1, PLA2G4A, MTOR, TIMP1, CREB1, CD163, EPHB2, ALOX12, DECR1

### Validation of specific target-chemical ingredient interactions by molecular docking

mTOR regulates the growth and metabolism of immune cells, as well as macrophage apoptosis (Zou et al., 2020). The therapeutic potential of mTOR inhibitors for COVID-19 has been reported (Fagone et al., 2020). Phospholipase A2 group IVA (PLA2G4A) is closely related to the arachidonic acid metabolic pathway, which can promote the degradation of phospholipids, and produce arachidonic acid, subsequently causing cytokine storm activation (Harizi et al., 2008). The “TCM component–active compound–specific target” networks analysis revealed that GRM1 and GRM5 could core specific proteins of LHQW, whereas mTOR and PLA2G4Awere potential core specific proteins of QFPD.

We next sought to determine which formulation was superior in treating mild-to-moderate COVID-19 with symptoms of depression (Asadi-Pooya & Simani, 2020; Havervall et al., 2021) or inflammation via targeting specific targets (GRM1, GRM5, mTOR and PLA2G4A). We performed molecular docking through GOLD and MOE to analyze the binding capacity between specific targets and the active compounds obtained from the ingredient–target network. ChemScore, ChemPLP, and binding energy values were applied to evaluate the docking results.

**FIGURE 5:**
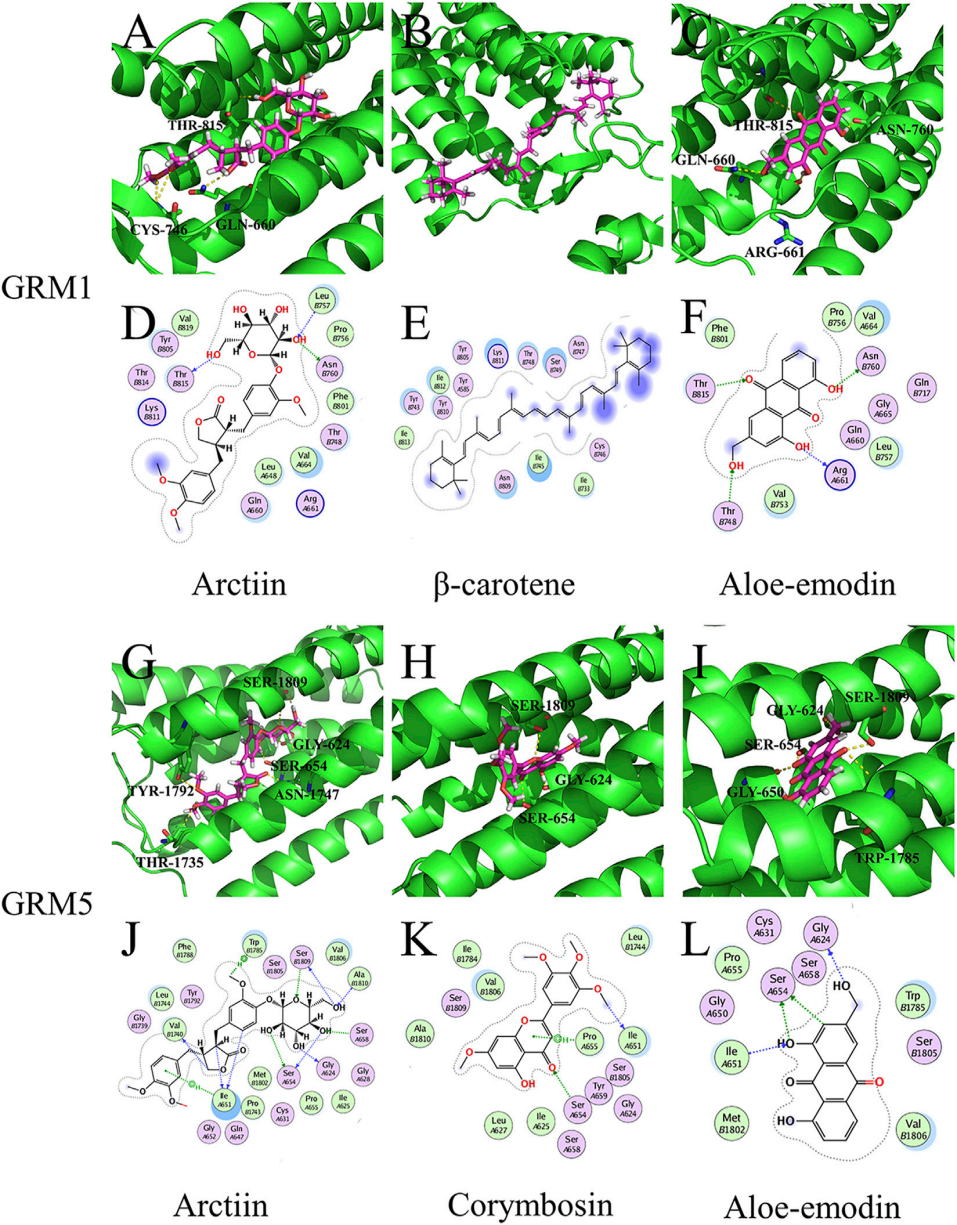
Molecular models of active compounds with specific key LHQW target genes. **(A)** Molecular model of arctiin, **(B)** β-carotene, **(C)** and aloe-emodin binding to GRM1. **(G)** Molecular model of arctiin, **(H)** corymbosin, **(I)** and aloe-emodin binding to GRM5. Yellow dashed lines represent H-bonds, thick pink sticks represent the active compounds, and thick green sticks represent residues in the protein-binding site. **(D)** Interactions between arctiin, **(E)** β-carotene, **(F)** aloe-emodin, and GRM1. **(J)** Interaction between arctiin, **(K)** corymbosin, **(L)** aloe-emodin, and GRM5. Blue dashed lines represent H-bonds between the active compounds and residue backbones, and green dashed lines represent H-bonds between the active compounds and residue sidechains. The dashed lines point to the acceptor.

Arctiin exhibited better binding activity than the controls ([11C]LY-2428703 and ADX-50938) when docked with GRM1 and GRM5 (Supplementary Tables S3, S4). It primarily formed three hydrogen bonds, each with THR-815, CYS-746, and GLN-660 residues on GRM1 (Figure 5A), and six hydrogen bonds with SER-1809, GLY-624, SER-654, ASN-1747, TYR-1792, and THR-1735 residues on GRM5 (Figure 5G). According to the target-chemical interactions, arctiin interacts with THR-815, LEU-757, and ASN-760 residues on GRM1 (Figure 5D) and TRP-1785, SER-1809, ALA-1810, SER-658, GLY-624, SER-654, ILE-651, and VAL-1740 residues on GRM5 (Figure 5J). In addition, β-carotene exhibited better binding activity than the control ([11C]LY-2428703) when docked with GRM1 (Supplementary Table S4). Other chemical ingredients also formed hydrogen bonds and interacted with different residues on specific targets (Figure 5).

**FIGURE 6:**
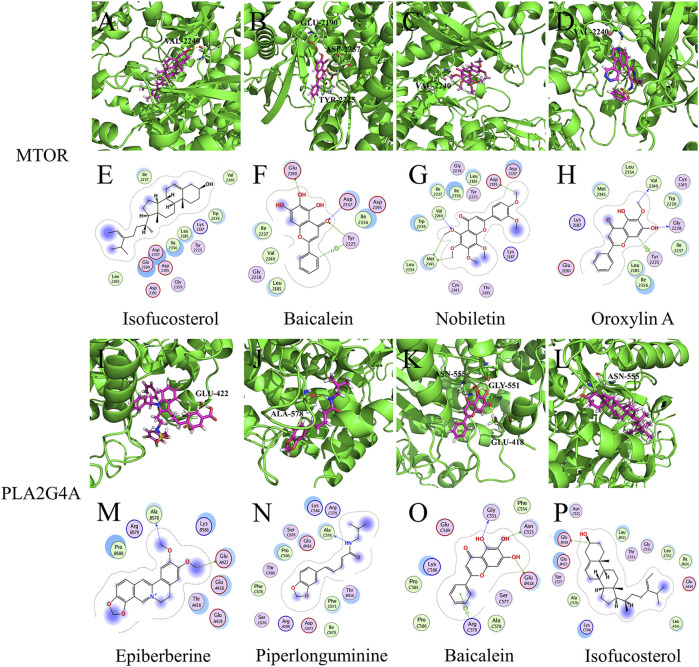
Molecular models of active compounds with specific QFPD target genes. **(A)** Molecular model of isofucosterol binding to mTOR. **(B)** Molecular model of baicalein, **(C)** nobiletin, **(D)** oroxylin A, **(I)** epiberberine, **(G)** piperlonguminine, **(K)** baicalein, **(L)** and isofucosterol binding to PLA2G4A. Yellow dashed lines represent H-bonds, thick pink sticks represent active compounds, and thick green sticks represent residues at the protein-binding site. **(E)** Interaction of isofucosterol, **(F)** baicalein, **(G)** nobiletin, and mTOR. **(H)** Interaction of oroxylin A, **(M)** epiberberine, **(N)** piperlonguminine, **(O)** baicalein, **(P)** isofucosterol, and PLA2G4A. Blue dashed lines represent H-bonds between the active compounds and residue backbones, and the green dashed lines indicate H-bonds between the active compounds and residue sidechains. The dashed lines point to the acceptor.

These results suggest that LHQW may be effective in treating psychological disorder symptoms in mild-to-moderate COVID-19 by targeting neurological disease-related targets (GRM1 and GRM5) through arctiin, corymbosin, and aloe-emodin. Meanwhile, QFPD is associated with inflammation and functions by targeting immunity- and inflammation-related factors (mTOR and PLA2G4A) through isofucosterol, baicalein, nobiletin, oroxylinA, epiberberine, and piperlonguminine (Figure 6, Supplementary Tables S5, S6).

### Validation of specific target-chemical ingredient interactions by molecular dynamic simulations

**FIGURE 7:**
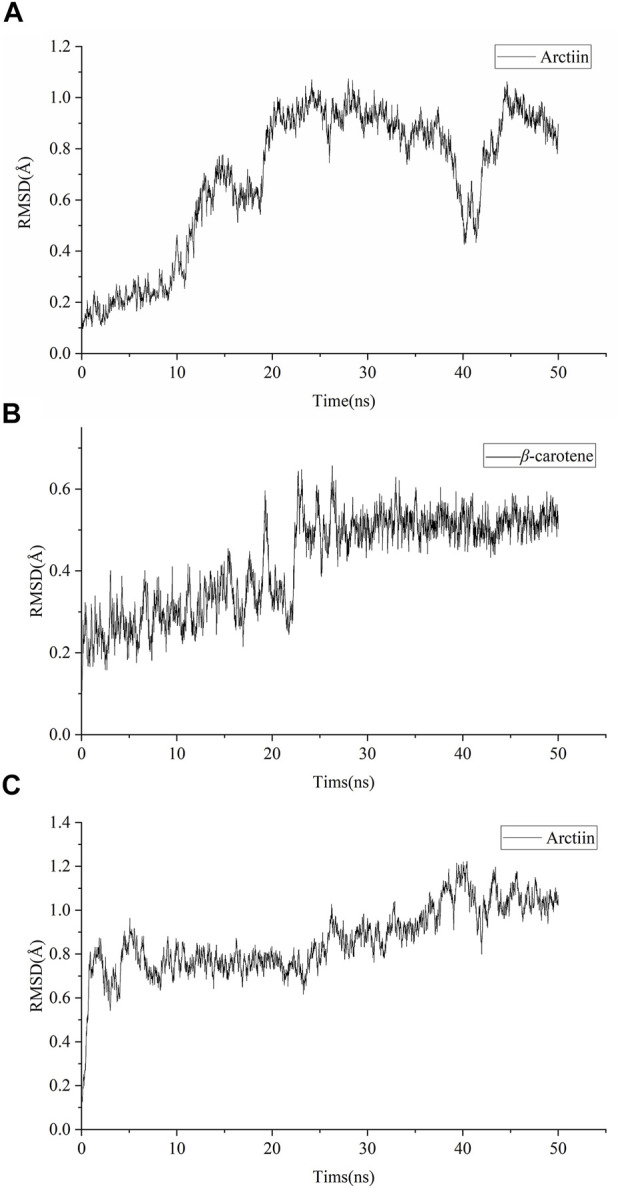
Root mean square deviation (RMSD) of molecular dynamic simulations. **(A)** RMSD of arctiin binding to GRM1. **(B)** RMSD of β-carotene binding to GRM1 **(C)** RMSD of arctiin binding to GRM5.

Amber18 was employed to analyze the binding capacity between specific targets (GRM1 and GRM5) and active compounds (arctiin and β-carotene) that demonstrated effects superior to the positive controls. The results of the target–ingredient binding (RMSD, RMSF, and SASA values) are shown in Figure 7 and Supplementary Figure S2, while those of the Gibbs free energy analyses are summarized in Table 2. RMSD can be used to characterize the stability of the active compounds that bind targets. The RMSD of arctiin binding to GRM1 is stable after 20 ns, with an RMSD value of approximately 0.9 Å at 20–35 ns (average RMSD = 0.69 Å; Figure 7A). β-carotene binding to GRM1 was stable at approximately 0.3 Å at 0–20 ns and approximately 0.5 Å at 25–50 ns, with an average RMSD value of 0.42 Å (Figure 7B). The RMSD of arctiin binding to GRM5 was stable after 5 ns, and the RMSD value was stable at approximately 0.7 Å at 5–25 ns and approximately 0.9 Å at 25–50 ns (average RMSD = 0.86 Å; Figure 7C). These data suggest that arctiin and β-carotene can bind stably with GRM1, and arctiin binds stably with GRM5. Hence, arctiin and β-carotene can stably bind to their targets, and may represent potential targeted drugs.

**TABLE 2:** Gibbs free energy of arctiin and β-carotene with GRM1 and GRM5.

Target protein	PDBID	Active compound	Gibbs free energy (kJ/mol)
GRM1	4OR2	Arctiin	-26.000
		β-carotene	-35.513
GRM5	6FFH	Arctiin	-37.330

LHQW, and QFPD, potential targets for COVID-19.

## Discussion

The outbreak and spread of the novel coronavirus disease, COVID-19, turned into a global public health emergency with serious socio-economic repercussions (Shen et al., 2021). Effective treatment of COVID-19 with allopathic medicine has faced several challenges, including its applicability to children and other specific subgroups of patients, as well as its relatively high cost (Ansems et al., 2021; Agarwal et al., 2022). Meanwhile, treatment with TCM can alleviate symptoms and prevent fatal deterioration of COVID-19 patients. As such, TCM has been incorporated into various COVID-19 treatment guidelines in China, thereby playing an indispensable role in its management (Li Q. et al., 2020; Huang et al., 2021).

LHQW and QFPD have shown efficacy in the treatment of mild-to-moderate COVID-19 cases. Indeed, LHQW has been widely used in China (Li L. C. et al., 2020). Clinically, LHQW has exhibited numerous advantages in treating COVID-19 (Runfeng et al., 2020; Hu et al., 2021). LHQW inhibits virus transmission and regulates immune function in influenza virus infections. In addition, QFPD exerts therapeutic effects via immune regulation, anti-infection and anti-inflammatory properties, and multi-organ protection in patients with mild-to-moderate COVID-19. In fact, the overall treatment efficacy rate for COVID-19 reportedly exceeds 90% (Luo et al., 2020; Wang et al., 2021).

According to clinical studies, an abnormal concentration of various cytokines (such as TNF-α, IL-6, IL-8) and an excessive release of reactive oxygen species in COVID-19 could cause an overactive immune response in COVID-19 (Behrens & Koretzky, 2017; Huang et al., 2020; Pedersen & Ho, 2020). More specifically, the continuous production of inflammatory factors can cause cytokine storm, which aggravates lung tissue damage and results in oxidative stress damage (Bettelli et al., 2006; Dai et al., 2018; Erlich et al., 2020). Quercetin and kaempferol present in LHQW can inhibit the overexpression of cytokines and alleviate multiple organ injuries in COVID-19 (Zhang et al., 2021). Similarly, QFPD contains active compounds capable of alleviating inflammation and preventing the initiation of the cytokine storm in COVID-19 by regulating multiple pathways (Zhao et al., 2021). Our research supports these findings as we show that LHQW and QFPD can regulate the immune system, reduce inflammation, and inhibit oxidative stress damage via related pathways, including those involved in immunity and inflammation, apoptosis, and viral infection, ultimately alleviating COVID-19 symptoms.

Although there are similarities between the two formulations, LHQW and QFPD have specific targets and contain different active compounds. In the present study, the active compounds in LHQW exhibited an excellent ability to bind to specific targets, namely GRM1 and GRM5. GRM1 and GRM5 are type I metabotropic glutamate receptors involved in various mental illnesses, including depression, anxiety, and cognitive impairment by regulating the function of neurotransmitters such as glutamate, dopamine, and norepinephrine (Pittaluga, 2016; Borbely et al., 2022). The long-term administration of two antidepressants (imipramine and citalopram) in mice inhibited the transcription of ionotropic glutamate receptors in multiple brain regions, suggesting that glutamate receptors are associated with depression (Boyer et al., 1998). Meanwhile, QFPD may play a role in COVID-19 treatment by targeting mTOR and PLA2G4A. As a serine-threonine kinase (Huang et al., 2020), decreased expression of mTOR can interfere with viral replication and protein synthesis.

Drugs that target the mTOR of Middle East respiratory syndrome coronaviruses are currently available (Kindrachuk et al., 2015). The “SARS-COV-2–human PPI network” constructed in this study, indicates that mTOR may be a potential key target of COVID-19, and its inhibitors may elicit curative anti-SARS-COV-2 effects (Gordon et al., 2020). Meanwhile, PLA2G4A can participate in arachidonic acid synthesis and produce large amounts of inflammatory factors, leading to cytokine storm initiation (Harizi et al., 2008). Current clinical studies have shown that the expression levels of inflammatory factors (IL-2, IL-7, IFN-γ, and TNF-α) positively correlate with the severity of COVID-19 (Huang et al., 2020), suggesting that PLA2G4A may be a key target in the development of COVID-19.

Our findings indicate that LHQW and QFPD may treat COVID-19 via pathways associated with inflammation, immunity, apoptosis, oxidative stress, etc. Meanwhile, the unique active compounds (Arctiin, Corymbosin, and Aloe-emodin) in LHQW target neurological disease-related genes (GRM1 and GRM5), and the special active compounds (isofucosterol, baicalein, nobiletin, oroxylin A, epiberberine, and piperlonguminine) in QFPD target immunity- and inflammation-related genes (mTOR and PLA2G4A). These indicated that LHQW might be suitable for mild-to-moderate COVID-19 with nervous system symptoms (Asadi-Pooya & Simani, 2020; Havervall et al., 2021) and QFPD may have a potential effect on regulating oxidative stress damage and inflammatory symptoms. In addition, the active compounds identified in this study can be further modified and optimized as candidate drugs for mild-to-moderate COVID-19.

However, this study has the following limitations, the active compounds are from TCMSP, and the assessment of relationships between the different compounds was insufficient. Hence, further studies should be performed to verify these results *in vitro* and *in vivo* employing, genomics, metabolomics, and proteomics approaches. Our study may be the basis for LHQW and QFPD in treating mild-to-moderate COVID-19, providing candidate active compounds for the clinical application of LHQW and QFPD.

## Data Availability

The datasets presented in this study can be found in online repositories. The names of the repository/repositories and accession number(s) can be found in the article/Supplementary Material.
