# Supplementary material for: Network pharmacology and computer-aided drug design to explored potential targets of Lianhua Qingwen and Qingfei Paidu decoction for COVID-19
Source: Front Pharmacol. 2022 Sep 23;13:1013428. doi: 10.3389/fphar.2022.1013428 (PMC9540507; doi:10.3389/fphar.2022.1013428)
Supplement: Supplementary file 3 [file Table3.docx]

Supplementary Material

# Supplementary Data

The active compounds of LHQW are in the attachment file. (Active compounds of LHQW.xls)

The active compounds of QFPD are in the attachment file. (Active compounds of QFPD.xls)

# Supplementary Figures and Tables

## Supplementary Figures

## Supplementary Figure 1


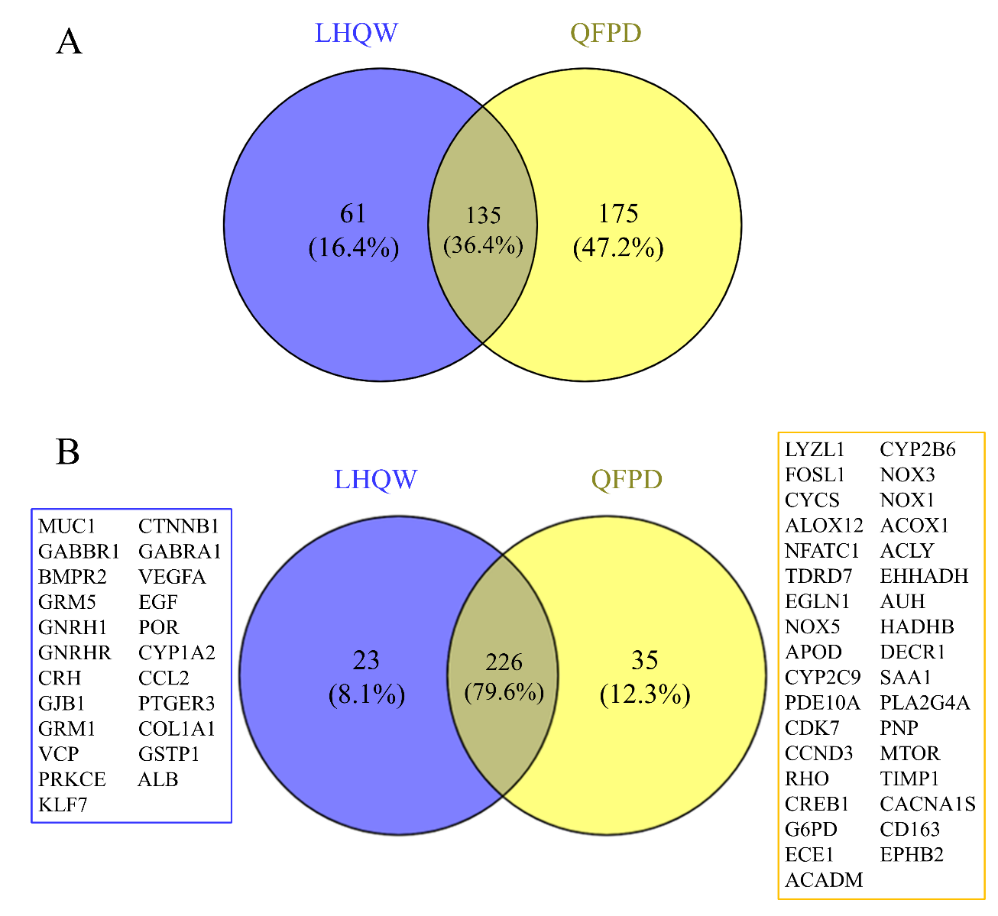


## Supplementary Figure 2


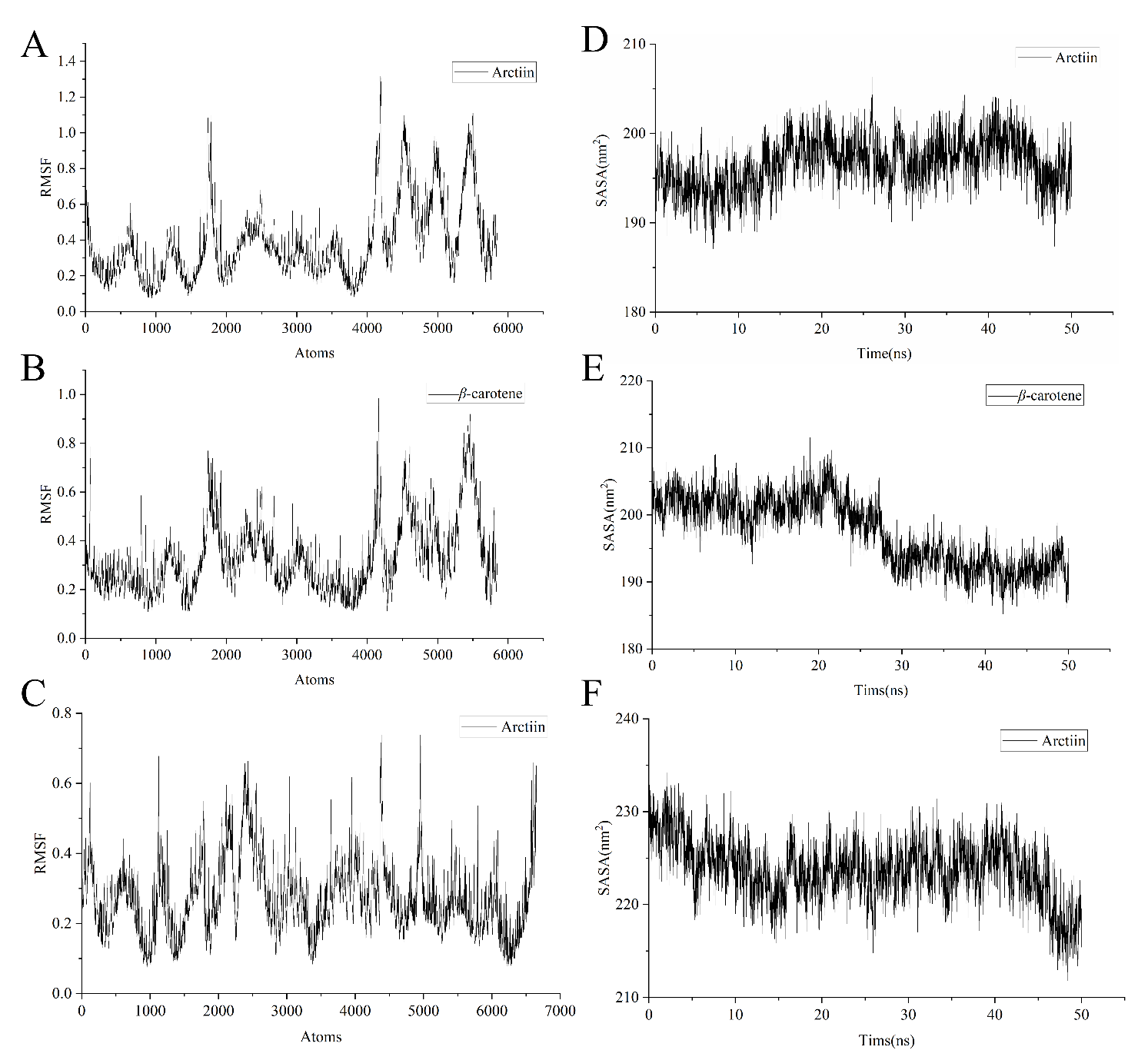


**Supplementary Figure 1.** Chemical ingredients and putative targets of the active compounds in *Lianhua Qingwen* (LHQW) and *Qingfei Paidu Decoction Qingfei Paidu Decoction* (QFPD). (A) Venn diagram of candidate ingredients in LHQW and QFPD. (B) Venn diagram of target genes in hosts of LHQW and QFPD.

**Supplementary Figure 2.** Root mean square fluctuation **(**RMSF) and solvent-accessible surface area (SASA) of Molecular dynamics simulations. (A) The RMSF of arctiin binding to GRM1. (B) The RMSF of β-carotene binding to GRM1. (C) The RMSF of arctiin binding to GRM5. (D) The SASA of arctiin binding to GRM1. (E) The SASA of *β*-carotene binding to GRM1. (F) The SASA of arctiin binding to GRM5.

## Supplementary Tables

Table S1. The Components, properties, and channel tropism of LHQW and QFPD.

| Components | Formulas | | Property and flavor | Channel tropism |
| --- | --- | --- | --- | --- |
| *Forsythiae Fructus*  (Lian Qiao) | LHQW |  | slightly cold, bitter | lung, heart, small intestine |
| *Glycyrrhizae Radix Et Rhizome*  (Gan Cao) | LHQW | QFPD | natured, sweet | heart, lung, spleen, stomach |
| *Lonicera Japonicae Flos*  (Jin Yin Hua) | LHQW |  | cold, sweet | lung, heart, stomach |
| *Pogostemonis Herba*  (Guang Huo Xiang) | LHQW | QFPD | slightly warm, pungent | spleen, stomach, lung |
| *Isatidis Radix*  (Ban Lan Gen) | LHQW |  | cold, bitter | heart, stomach |
| *Armeniacae Semen Amarum*  (Ku Xing Ren) | LHQW | QFPD | slightly warm, bitter | lung, large intestine |
| *Houttuyniae Herba*  (Yu Xing Cao) | LHQW |  | slightly cold, pungent | lung |
| *Ephedrae Herba*  (Ma Huang) | LHQW | QFPD | warm, pungent, slightly bitter | lung, urinary bladder |
| *Dryopteridis Crassirhizomatis Rhizoma* (Mian Ma Guan Zhong) | LHQW |  | slightly cold, bitter | liver, stomach |
| *Rhei Radix Et Rhizoma*  (Da Huang) | LHQW |  | cold, bitter | spleen, stomach, large intestine, liver, cardiovascular |
| *Menthae Haplocalycis Herba*  (Bo He) | LHQW |  | cold, pungent | lung, liver |
| Gypsum Fibrosum  (Shi Gao) | LHQW | QFPD | sweet | lung, heart |
| *Rhodiolae Crenulatae Radix Et Rhizoma*  (Hong Jing Tian) | LHQW |  | slightly cold, bitter | lung, heart, Small intestine |
| *Cinnamomi Ramulus*  (Gui Zhi) |  | QFPD | warm, pungent and sweet | Heart, lung and bladder |
| *Alismatis Rhizoma*  (Ze Xie) |  | QFPD | light and cold, sweet | Kidney and bladder |
| *Polyporus*  (Zhu Lin) |  | QFPD | light and flat, sweet | Kidney and bladder |
| *Atractylodis Macrocephalae Rhizoma*  (Bai Zhu) |  | QFPD | warm, bitter and sweet | Spleen and stomach |
| Poria  (Fu Lin) |  | QFPD | light and flat, sweet | Heart, lung, spleen and kidney |
| *Bupleuri Radix*  （Chai Hu） |  | QFPD | slightly cold and bitter | Liver, gallbladder and lung |
| Astragali Radix  (Huang Qin) |  | QFPD | cold, bitter | Lung, gallbladder, spleen, large intestine and small intestine |
| *Pinelliae Rhizoma Praeparatum Cum Zingibere Et Alumine*  (Jiang Ban Xia) |  | QFPD | warm, pungent | Spleen, stomach and lung |
| *Zingiberis Rhizoma Recens*  (Sheng Jiang) |  | QFPD | mild temperature and pungent | Lung, spleen and stomach |
| *Asteris Radix Et Rhizoma*  (Zi Wan) |  | QFPD | warm and bitter | lung |
| *Farfarae Flos*  (Dong Hua) |  | QFPD | Warm. pungent and slightly bitter | lung |
| *Belamcandae Rhizoma*  (She Gan) |  | QFPD | cold, bitter | lung |
| *Asari Radix Et Rhizoma*  (Xi Xin) |  | QFPD | warm, pungent, | Heart, lung and kidney |
| *Dioscoreae Rhizoma*  (Shan Yao) |  | QFPD | flat, sweet | Spleen, lung and kidney |
| *Aurantii Fructus Immaturus*  (Zhi Shi) |  | QFPD | slightly cold, bitter, pungent, and sour | Spleen and stomach |
| *Citri Reticulatae Pericarpium*  (Chen Pi) |  | QFPD | warm, bitter, and pungent | Lung and spleen |

Table S2. Number of active compounds and genes in LHQW and QFPD.

| Formulas | Number of active compounds | Number of genes in hosts |
| --- | --- | --- |
| LHQW | 196 | 249 |
| QFPD | 310 | 261 |

Table S3. The chemical structures contained from LHQW participating in docking.

| **Target protein** | **Active compound** | **Chemical structure** |
| --- | --- | --- |
| GRM1 | CFMTI | 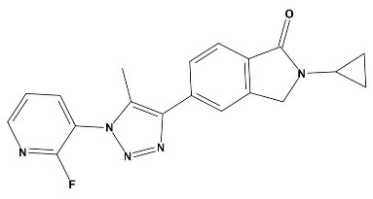 |
|  | [11C]LY-2428703 | 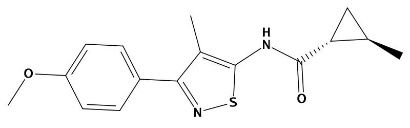 |
|  | Arctiin | 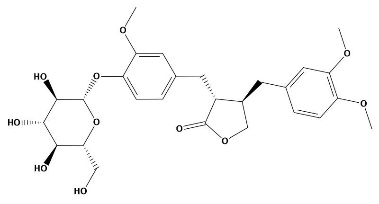 |
|  | *β*-carotene | 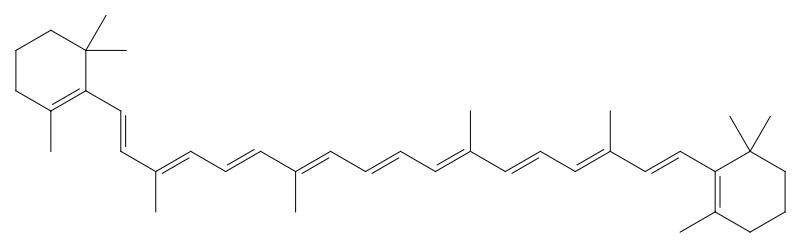 |
|  | Aloe-emodin | 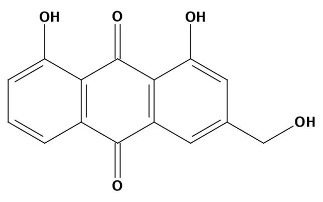 |
| GRM5 | HTL-14242 | 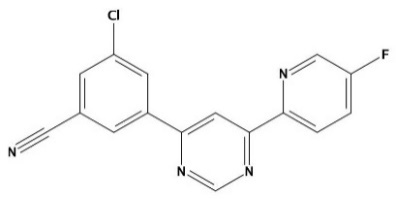 |
|  | ADX-50938 | 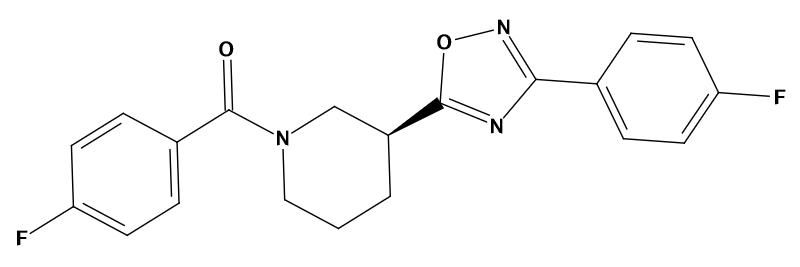 |
|  | Arctiin | 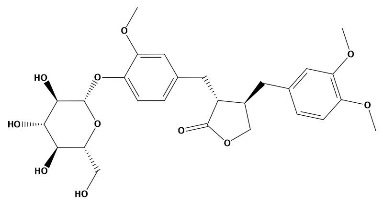 |
|  | Corymbosin | 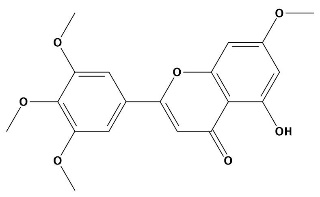 |
|  | Aloe-emodin | 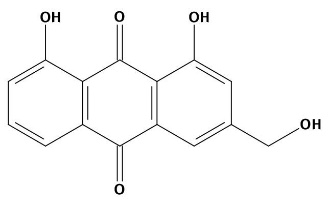 |

Table S4. Docking score of chemical ingredients with specific targets of LHQW.

| **Target protein** | **PDBID** | **Active**  **compound** | **Source** | **ChemPLP** | **ChemScore** | **Affinity**  **(kcal/mol)** |
| --- | --- | --- | --- | --- | --- | --- |
| GRM1 | 4OR2 | CFMTI | Control | 88.87 | 35.91 | -9.07 |
|  |  | [11C]LY-2428703 | Control | 73.16 | 44.77 | -8.53 |
|  |  | Arctiin | Lian Qiao | 81.58 | 18.8 | -10.51 |
|  |  | *β*-carotene | Jin Yin Hua | 79.64 | 34.17 | -10.16 |
|  |  | Aloe-emodin | Da Huang  Bo He | 72.68 | 48.57 | -6.73 |
| GRM5 | 6FFH | HTL-14242 | Control | 103.06 | 38.50 | -6.52 |
|  |  | ADX-50938 | Control | 89.36 | 36.82 | -6.91 |
|  |  | Arctiin | Lian Qiao | 93.75 | 34.26 | -6.29 |
|  |  | Corymbosin | Jin Yin Hua | 77.47 | 28.92 | -7.65 |
|  |  | Aloe-emodin | Da Huang  Bo He | 64.58 | 21.31 | -5.39 |

Table S5. The chemical structures contained from QFPD participating in docking

| **Target protein** | **Active compound** | **Chemical structure** |
| --- | --- | --- |
| **MTOR** | Apitolisib | **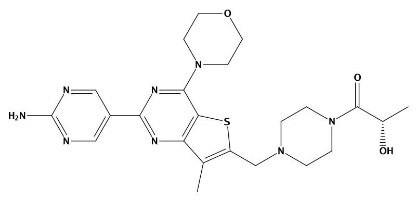** |
|  | Gedatolisib | **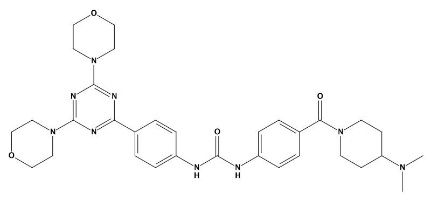** |
|  | Isofucosterol | **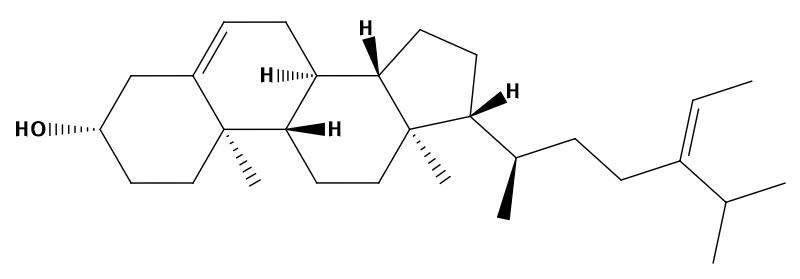** |
|  | Baicalein | **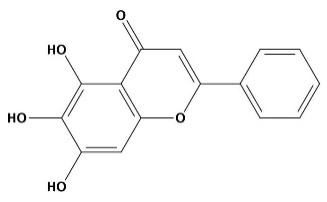** |
|  | Nobiletin | **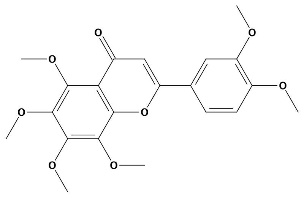** |
|  | Oroxylin A | **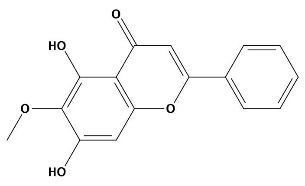** |
| **PLA2G4A** | YM-26567-1 | **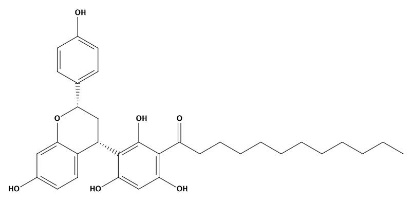** |
|  | BMS-181162 | **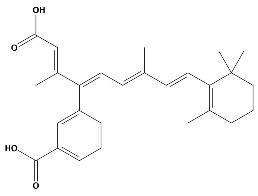** |
|  | Epiberberine | **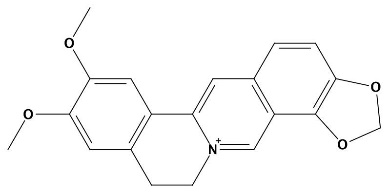** |
|  | Piperlonguminine | **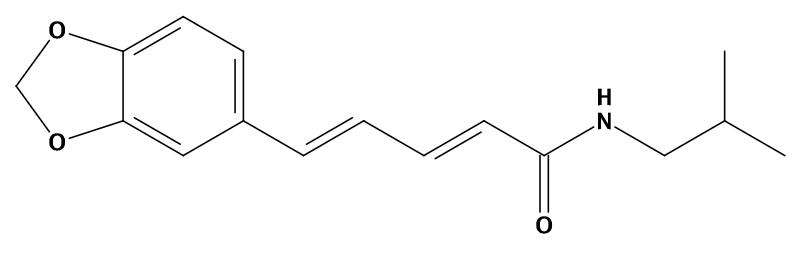** |
|  | Baicalein | **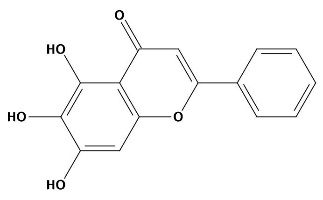** |
|  | Isofucosterol | **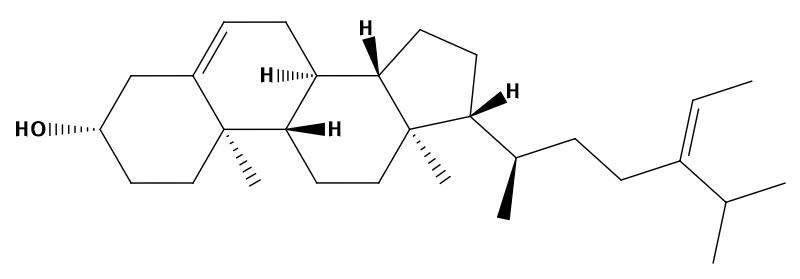** |

Table S6. Docking score of chemical ingredients with specific targets of QFPD.

| **Target protein** | **PDBID** | **Active**  **compound** | **Source** | **ChemPLP** | **ChemScore** | **Affinity**  **(kcal/mol)** |
| --- | --- | --- | --- | --- | --- | --- |
| MTOR | 4JT6 | Apitolisib | Control | 97.22 | 36.75 | -8.52 |
|  |  | Gedatolisib | Control | 94.44 | 30.25 | -10.71 |
|  |  | Isofucosterol | Shan Yao | 84.99 | 40.38 | -6.59 |
|  |  | Baicalein | Huang Qin  Ban Xia | 74.05 | 34.91 | -6.60 |
|  |  | Nobiletin | Zi Wan  Zhi Shi | 70.95 | 28.81 | -8.21 |
|  |  | Oroxylin A | Huang Qin | 70.68 | 32.26 | -7.04 |
| PLA2G4A | 1CJY | YM-26567-1 | Control | 92.08 | 26.14 | -9.06 |
|  |  | BMS-181162 | Control | 73.28 | 22.10 | -8.22 |
|  |  | Epiberberine | Huang Qin | 64.99 | 22.38 | -6.76 |
|  |  | Piperlonguminine | Shan Yao | 63.97 | 23.86 | -6.33 |
|  |  | Baicalein | Huang Qin | 62.14 | 24.18 | -6.64 |
|  |  | Isofucosterol | Shan Yao | 60.12 | 24.83 | -7.87 |
